# Ecological factors affecting prosociality in marmoset monkeys

**DOI:** 10.1007/s10071-026-02049-1

**Published:** 2026-02-10

**Authors:** Anand R. Mysorekar, Anushka Vispute, Cory T. Miller

**Affiliations:** Cortical Systems and Behavior Laboratory, University of California, San Diego, United States

**Keywords:** Marmoset, Prosocial, Cooperation, Food-sharing, Social decision-making, Ecology

## Abstract

Prosocial behaviors, such as cooperation and food sharing, are critical for maintaining group cohesion in social species, yet the influence of transient physiological states on these behaviors remains poorly understood. This study investigates how short-term ecological factors impact social behavior in common marmosets (*Callithrix jacchus*), a highly prosocial nonhuman primate species. Specifically, we tested how food divisibility (small vs. large food items) and time since food access (TSFA; 0 h, 1 h, and 3 h) influenced food sharing behavior. Results revealed that larger food portions consistently promoted prosocial interactions, while longer durations since food access shifted behavior toward individualism. No significant interaction effect between TSFA and food size was observed. These findings suggest that marmoset prosociality is sensitive to immediate ecological conditions, reflecting flexible, context-dependent social decision-making.

## Introduction

Understanding the conditions that shape prosocial behavior is central to unraveling the evolution of cooperation in social species (de Waal 2008; Jaeggi and Gurven 2013a; Burkart et al. 2014). Prosociality refers to behaviors that benefit another individual, such as helping, tolerating access, or sharing, and are often found in species in which individuals in a social group are heavily dependent on each other (Apps et al. 2016; Burkart and van Schaik 2020). In Callitrichid primates, including common marmosets (Callithrix jacchus), these behaviors are embedded within a cooperative breeding system in which non-breeding helpers contribute to infant care through provisioning, transport, and protection (Brown et al. 2005; Burkart et al. 2007). One prominent expression of prosociality in marmosets is food sharing, defined as the voluntary transfer of food or tolerance of another’s access, which includes allofeeding, co-feeding, and conflict-free sequential use (Burkart et al. 2007; Silk et al. 2013; Jaeggi and Gurven 2013b). While long-term social factors such as kinship, pair bonding, and established social roles influence prosocial tendencies (Silk et al. 2013), recent studies suggest that immediate ecological and physiological states may have significant short-term effects on cooperative decision-making, including food-sharing (Mustoe et al. 2015; Yamada 2017).

Although not all mammalian species engage in food sharing, this prosocial behavior is not unique to callitrichids (Jaeggi and Gurven 2013a; Burkart et al. 2014). Among apes, food sharing in both chimpanzees and bonobos reflects reciprocity, harassment avoidance, or social bond maintenance (Silk et al. 2013; Wittig et al. 2014). In non-primate species, such as wolves, meerkats, and vampire bats, food sharing supports kin-selected provisioning, cooperative foraging, or reciprocal exchange (Wilkinson 1984; Mech 1999; Clutton-Brock et al. 2001). Within this comparative landscape, however, marmosets do stand out for the frequency, spontaneity, and low-conflict nature of their food sharing. Unlike other primates, marmosets routinely share food without solicitation, even under minimal social pressure (Burkart and van Schaik 2020). This uniquely tolerant social profile makes them a powerful model for examining the flexibility and ecological sensitivity of prosociality.

The prosocial tendencies of marmosets reflect adaptations that evolved in response to the heterogeneous and seasonally dynamic Atlantic Forest of northeastern Brazil in which the species is endemic (Rylands and de Faria 1993; Miller 2017; Schiel and Souto 2017; De la Fuente et al. 2022). Their primary food sources, such as tree gums, fruits, and insects, are patchily distributed and seasonal, often requiring specialized foraging behaviors (De la Fuente et al. 2014; Abreu et al. 2016; Garber et al. 2020; Ngo et al. 2022). In addition, the energetic demands of raising twins within a cooperative breeding system make group cohesion and coordinated provisioning particularly important in marmosets (Digby and Barreto 1993; Schiel and Huber 2006; Saltzman et al. 2009). This socioecological environment is believed to promote high tolerance around resources, including frequent and spontaneous food sharing (Burkart et al. 2007; Mustoe et al. 2015). Despite substantial work on long-term determinants of marmoset cooperative behavior, far less is known about how short-term ecological factors, such as hunger state or resource divisibility, modulate prosocial decision-making. Here, we address this gap by experimentally manipulating food size and time since food access (TSFA) to test how acute ecological conditions influence food-sharing behavior in common marmosets.

## Methods

### Subjects

This study involved 29 common marmosets (*Callithrix jacchus*): 12 males (4 parents, 8 offspring) and 17 females (6 parents, 11 offspring) housed across six different family groups (Table 1).

**Table 1:** Family group composition of marmosets

Group	Parents (M, F)	Offspring (M, F)	Total Animals
1	(1, 1)	(1, 1)	4
2	(0, 1)	(2, 2)	5
3	(1, 1)	(1, 2)	5
4	(1, 1)	(0, 2)	4
5	(1, 1)	(3, 1)	6
6	(0, 1)	(1, 3)	5

All offspring were juveniles or subadults (approximately 1.5-3 years old); no infants or recently weaned individuals were present in any group. Because family group size and composition can influence food sharing, behavior was analyzed as proportions within each session. This controls for variations in group size and allows for comparisons across groups. All animals were kept in enriched enclosures on a 12-hour light/dark cycle, with ad libitum access to water and a controlled diet provided outside experimental sessions. All experimental procedures were approved by the Institutional Animal Care and Use Committee at the University of California, San Diego.

Data collection was conducted in two phases. During the first phase, animals in three family groups were tested under the experimental paradigms outlined below. After completing testing in these groups, a method for individual identification was introduced to enable analysis of individual, age, and sex differences. In the second phase, three additional groups were tested using this identification method. Subjects’ hair tufts were temporarily dyed using Arctic Fox Semi-Permanent Hair Color (vegan, cruelty-free, and non-toxic) in unique combinations to allow for identification in video recordings. Because individual identification was only available for the second phase, sex and age information was limited to half of the dataset. As such, sex and age could not be included as fixed effects in the main models without substantial data loss. Instead, sex and age effects were analyzed separately in the subset of animals for which this information was available.

## Experimental design and procedure

We used a within-group design to investigate the effects of food size and time since food access (TSFA) on prosocial behavior. Each group was exposed to three TSFA conditions (0 h, 1 h, and 3 h) and presented with two food sizes (small and large). Testing was conducted in a shared marmoset colony room, where additional, non-subject marmosets were present in separate enclosures without physical access to the test box, reflecting the animals’ typical social environment.

Sessions were conducted at approximately the same time each day in the afternoon, and were not scheduled specifically to coincide with known peak feeding periods, to minimize variability due to circadian rhythms. TSFA was operationalized by temporarily removing access to routine food items for 0, 1, or 3 h prior to testing. Animals had access to their regular diet outside of the specified TSFA interval and were not without food beyond the designated restriction period.

The food used was Strawberry Bio-Serve Electro-Gel (Electrolyte Replenisher), a thermoreversible gel containing balanced concentrations of electrolytes and glucose, commonly used as dietary enrichment for marmosets. Food size was manipulated by presenting large portions as one-fourth of a full gel unit and small portions as one-sixteenth. These sizes were based on prior lab observations indicating they were sufficient for simultaneous consumption by multiple monkeys, enabling food sharing.

**Fig. 1:**
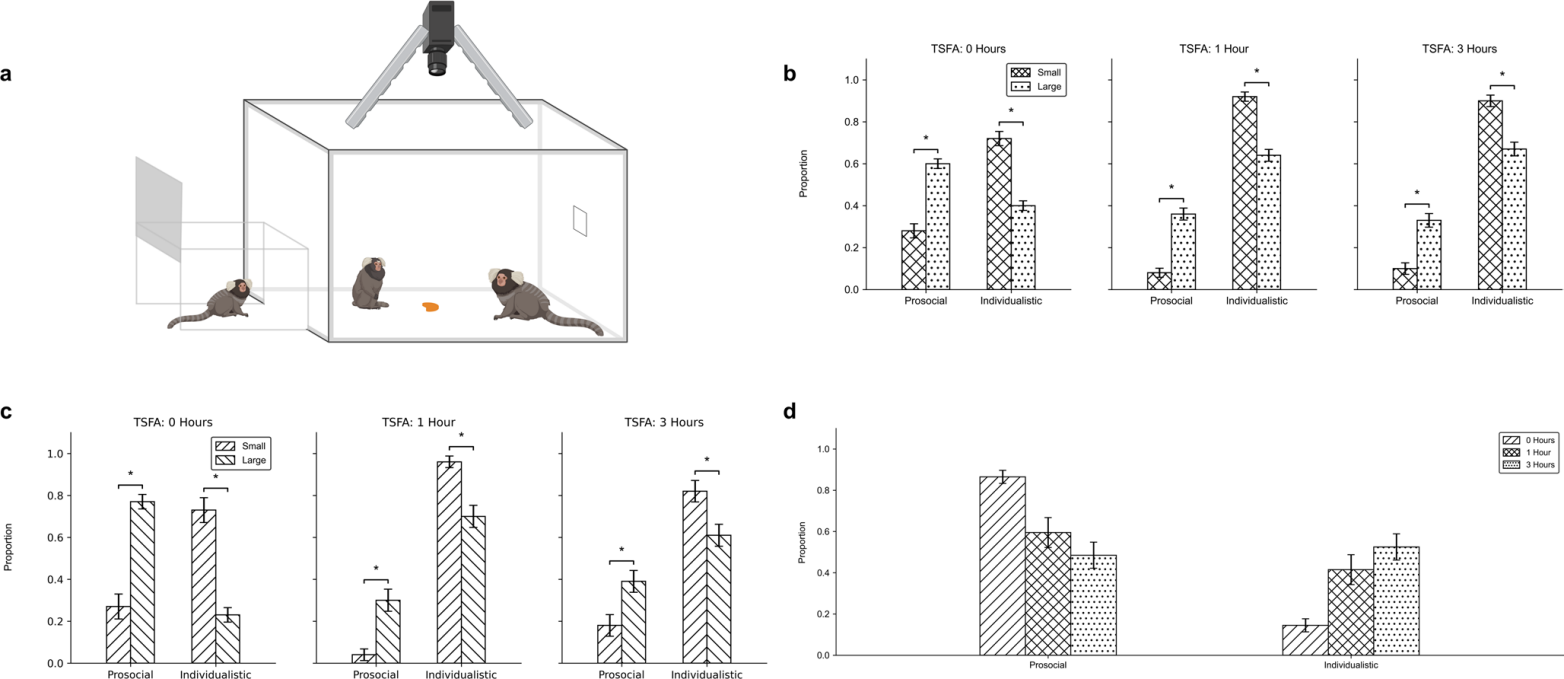
Effects of food size and time since food access (TSFA) on marmoset prosocial behavior. (**a**) Experimental setup: trials occurred in a test box connected to the home cage. Monkeys could freely enter the test box through the entrance tunnel once the door was opened. An overhead camera recorded all sessions. Food was placed at a randomized location in each trial. (**b**) Session-level behavioral proportions across food sizes and TSFA conditions in the full dataset, analyzed using a repeated-measures ANOVA with food size and TSFA as within-group factors (small food: crosshatch; large food: dotted). (**c**) Same comparison as in panel b for a restricted dataset including session-level behavioral proportions from the three family groups for which individual identification was available and that participated in all three TSFA conditions with both food sizes, analyzed using the same repeated-measures ANOVA approach (small food: forward slash; large food: backslash). This subset represents half of the total dataset (*n* = 54 sessions) and includes both parents and offspring and both males and females. (**d**) Behavioral proportions across TSFA conditions from a single representative group (0 h: forward slash; 1 h: crosshatch; 3 h: dotted). Significance markers are indicated with asterisks (*p* < 0.05)

The test box measured 61 cm × 47.75 cm × 47.75 cm and was constructed from plexiglass and 80/20 T-Slotted extrusions, allowing for unrestricted visual and physical interaction between subjects. An overhead camera provided a full view of the test box floor for later behavioral coding. Figure 1a illustrates this setup.

Prior to data collection, animals were habituated to the testing setup over multiple sessions in which the test box was accessible without food presentation or TSFA manipulation. During habituation, groups were allowed to freely explore the test box and entrance tunnel. No systematic differences in willingness to enter the test box were observed across groups and, while individual participation varied, no specific class of animals consistently avoided the test box.

Each group completed 18 test sessions, with six sessions conducted under each TSFA condition. Each session consisted of five trials, all conducted under the same TSFA condition. Across sessions, each TSFA condition was presented 30 times per group (15 small and 15 large food trials), for a total of 90 trials per group and 540 trials across all six groups. Within a session, food size varied across trials and was randomized, such that across all sessions for a given TSFA condition, food sizes were evenly distributed. To prevent operator bias, the placement of the food item was randomized each trial.

At the start of each trial, the door separating the cage from the entrance tunnel was opened, allowing monkeys to move freely between the test box and their home enclosure. A five-minute trial began immediately after the door was opened and continued until one of the following occurred: the five-minute period ended, the food was fully consumed, or a monkey removed the food and brought it into the home enclosure. At the end of each trial, the door was closed, and a five-minute inter-trial interval allowed subjects to consume the food and helped reduce potential carryover effects such as lingering satiety.

## Behavioral coding and measures

Videos were analyzed post-experiment to identify distinct behaviors exhibited during each trial. Because no standardized ethogram existed for short-term food sharing in marmosets, we developed one through direct observation of the sessions. While some behaviors (e.g., co-feeding and tolerant sharing) are categorized in the literature as social tolerance rather than prosociality, here we included them under a broader prosocial category. Our rationale is that these behaviors reflect the absence of competition and allow shared or sequential access to food, which are socially meaningful outcomes distinct from purely individualistic feeding. For clarity, ‘nearby’ was defined as within one body length of the animal currently interacting with the food and in a position to access the food if conflict occurred.

All behavioral occurrences by any group member were coded continuously over the full 5-minute duration of each trial using this ethogram (Table 2). Each behavior was recorded as a discrete event upon onset rather than as a duration-based measure. Behaviors were categorized as prosocial, individualistic, or neutral and summarized at the session level by computing the proportion of prosocial behaviors relative to the total number of prosocial and individualistic behaviors observed within that session; the complementary proportion reflected individualistic behavior. Two trained observers independently coded the data: one for the first three groups and the other for the last three. Inter-observer reliability was high, with an intraclass correlation coefficient (ICC, model 2,1) of 0.95 (95% CI: [0.9497, 0.9500], *p* < 0.001) indicating excellent agreement.

**Table 2:** Categorization of observed behaviors into prosocial, individualistic, and neutral groups based on food sharing interactions

Behavior Category	Behavior Name	Description
Prosocial	Food Sharing	Two or more monkeys eat simultaneously, without conflict
Prosocial	Tolerant Sharing	One monkey eats while another is nearby, without conflict or competition
Prosocial	Sequential Engagement	Monkeys take turns eating; one stops to allow another to eat before resuming
Prosocial	Allofeeding	One monkey directly transfers food to another, either mouth-to-mouth or by offering a portion
Individualistic	Solitary Consumption	One monkey eats alone, with no interaction from others nearby
Individualistic	Snatching	One monkey takes the food and immediately retreats into the home cage
Individualistic	Contest	Two or more monkeys engage in aggressive behavior over the food (e.g., fighting)
Neutral	Non-Participation	Monkeys do not interact with the food or engage in any food-related behavior during the trial

### Data analysis

Data were organized using Microsoft Excel and processed in Python, and statistical analyses were conducted with the pingouin package. Repeated-measures ANOVAs were used to assess the main and interaction effects of food size and TSFA on session-level behavioral proportions. Type II sums of squares were used, consistent with our focus on main effects. To address incomplete individual participation across conditions, we conducted a secondary analysis with a strict inclusion criterion. All statistical tests were two-tailed with α = 0.05.

To assess whether trial order within a session influenced behavior, we conducted an additional trial-level analysis using a linear mixed-effects model. Trial number was included as a fixed effect, with ‘group’ specified as a random intercept to account for repeated measurements within groups. This analysis was used to evaluate potential order effects while controlling for the hierarchical structure of the data.

## Results

A repeated-measures ANOVA on the full dataset (Fig. 1b) revealed a significant effect of food size on prosocial behavior (F(1,5) = 9.44, p = 0.028). Across all TSFA conditions, monkeys exhibited more prosocial behavior with large food portions and more individualistic behavior with small portions. The ANOVA also revealed a significant effect of TSFA (F(2,10) = 9.13, p = 0.006), indicating that overall behavior varied with hunger state. Specifically, prosocial behavior tended to decline, and individualistic behavior increased as the time since last food access lengthened. No significant interaction between food size and TSFA was observed (p = 0.099, Greenhouse-Geisser corrected), though the uncorrected value was marginal (p = 0.057), suggesting only a weak trend.

To ensure that these effects were not driven by uneven participation across conditions, we conducted a secondary analysis using a strict inclusion threshold that required monkeys to have participated in all three TSFA conditions and both food sizes. In this restricted dataset (Fig. 1c), the ANOVA confirmed a significant effect of TSFA (F(2,4) = 13.52, *p* = 0.017), but not food size (*p* = 0.21) or the interaction (*p* = 0.20). This indicates that TSFA was the more robust predictor of behavior under balanced participation criteria.

To further examine the effect of TSFA, we conducted pairwise comparisons between the three time conditions. None of the contrasts were significant after correction. However, uncorrected tests indicated a significant difference between 0 and 3 h (*p* = 0.045) and a marginal difference between 0 and 1 h (*p* = 0.052), while the 1 vs. 3-hour contrast was non-significant (*p* = 0.88). These uncorrected trends suggest a gradual behavioral shift across TSFA conditions rather than a stepwise change. Figure 1d shows a representative group in which prosocial behavior declined, and individualistic behavior increased with greater TSFA. While this pattern was observed in some groups, others remained stable across conditions, likely contributing to the lack of corrected pairwise effects.

To evaluate whether trial order within a session influenced behavior, we reanalyzed the data at the trial level using a linear mixed-effects model with ‘group’ as a random intercept. Trial number showed a statistically reliable but modest positive effect (β = 0.033, *p* < 0.001), indicating that later trials were slightly more prosocial than earlier ones. Importantly, the main effects of TSFA and food size remained significant and in the same direction when controlling for trial number, and no significant interactions emerged. This confirms that our conclusions are robust and not dependent on trial order.

In addition, exploratory analyses were conducted on the subset of animals for which individual identification was available to assess potential effects of age and sex on prosocial behavior. These analyses did not reveal significant main effects of age or sex, nor interactions with food size or TSFA. Given that individual-level demographic information was available for only half of the dataset, age and sex were not included as factors in the primary analyses.

## Discussion

We found that marmosets were more likely to share food when portions were large, indicating that resource abundance promotes prosocial behavior. However, during longer TSFA durations, individuals became more self-serving, suggesting that acute hunger reduces cooperation in socially competitive contexts. This aligns with prior findings that resource abundance promotes prosociality. Wittig et al. (2014), for example, found that chimpanzees were more likely to share food when resources were plentiful. Our findings similarly suggest that marmoset food sharing is context-dependent, increasing with larger portions rather than being automatic or unconditional. A key distinction is that while previous studies often emphasize long-term social structures, such as kinship or reciprocity, our results highlight the role of short-term ecological variables, specifically food size and time since food access, as immediate drivers of cooperative behavior.

The findings complement prior work showing that hunger can have significant impacts on decision-making in marmosets. Carvalho et al. (2022) showed that food deprivation increased choice consistency in an individual, non-social foraging task, an outcome interpreted as improved “decision-making efficiency”. Specifically, animals were more reliable in selecting higher-value options and less prone to variable or impulsive responding (Carvalho et al. 2022). Importantly, the present study differs fundamentally in task demands because feeding decisions occurred in a social context, where behavior is shaped not only by only considering immediate caloric gain but also by tolerance, competition, and access to resources in the presence of group members. In this context, labelling prosocial versus individualistic behavior as more or less “efficient” is less straightforward because prosocial sharing may carry longer-term benefits (e.g., maintaining tolerance, reinforcing social relationships, etc.) that are not captured by short-term food intake. Whereas hunger in solitary tasks may sharpen valuation and reduce variability, our results suggest that hunger in social contexts shifts how competing demands may be weighed, biasing behavior toward short-term strategies when resources are contested. Together, these findings support a broader view that the behavioral effects of hunger are context-dependent, enhancing consistent value-based choice when social consequences are minimal, but reducing prosocial tolerance when feeding occurs in a more competitive social landscape.

Our results underscore the importance of both external resource conditions and internal physiological states in shaping cooperative behavior. Larger food portions increased prosocial behavior, while acute hunger appeared to diminish it. This interaction suggests that social decision-making is flexible and influenced by immediate ecological constraints. Neurobiologically, these results motivate future studies investigating how hunger modulates neural circuits involved in reward, impulse control, and social cognition, such as the orbitofrontal cortex and anterior cingulate cortex (Apps et al. 2016).

This study provides novel evidence that prosocial behavior in marmosets is not fixed, but dynamically shaped by short-term ecological and physiological variables. By jointly manipulating food portion size and time since last food access, we demonstrate that cooperation is promoted by resource abundance but suppressed by acute hunger, highlighting the context sensitivity of social behavior even in a highly cooperative species. More broadly, these findings lay the groundwork for future investigations into the neural and hormonal mechanisms that mediate the interplay between internal states and social behavior (Lefevre et al. 2024), advancing our understanding of the neurobiological foundations of cooperation.

## Data Availability

Code and aggregated analysis files are available from the corresponding author upon request.
